# Supplementary figures and images for: A new branched proximity hybridization assay for the quantification of nanoscale protein–protein proximity
Source: PLoS Biol. 2019 Dec 11;17(12):e3000569. doi: 10.1371/journal.pbio.3000569 (PMC6905527; doi:10.1371/journal.pbio.3000569)

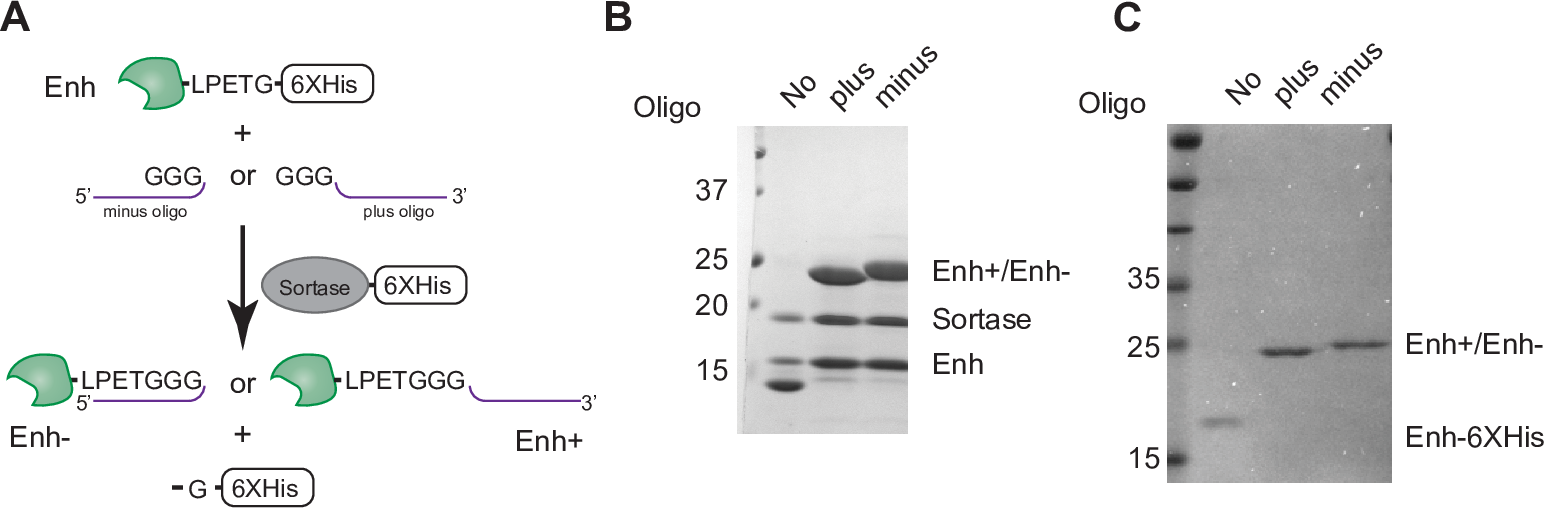

Supplement: S1 Fig — (A) Schematic presentation of the labeling reaction. (B and C) Coomassie-stained 12.5% reducing SDS-PAGE gel showing the composition of materials after sortase-mediated transpeptidation (B) or after further clearance with Ni-NTA column (C). bPHA, branched proximity hybridization assay; Enh, Enhancer; Ni-NTA; nickel-nitrilotriacetic acid. (TIF) [file pbio.3000569.s001.tif]

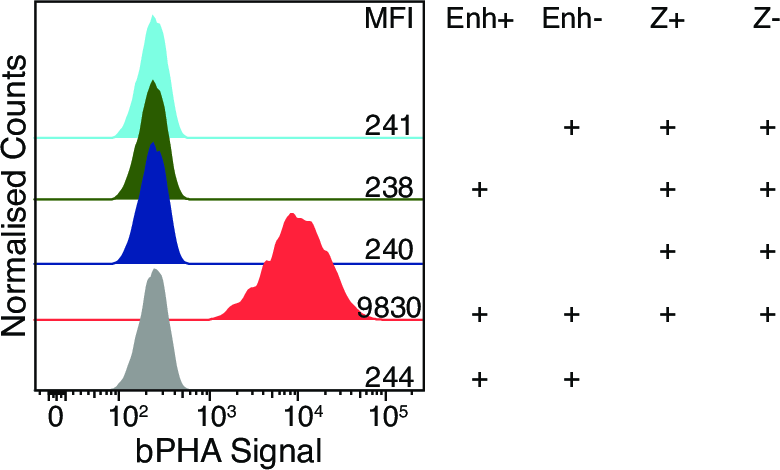

Supplement: S2 Fig — bPHA, branched proximity hybridization assay; Enh, Enhancer; GFP; green fluorescent protein. (TIF) [file pbio.3000569.s002.tif]

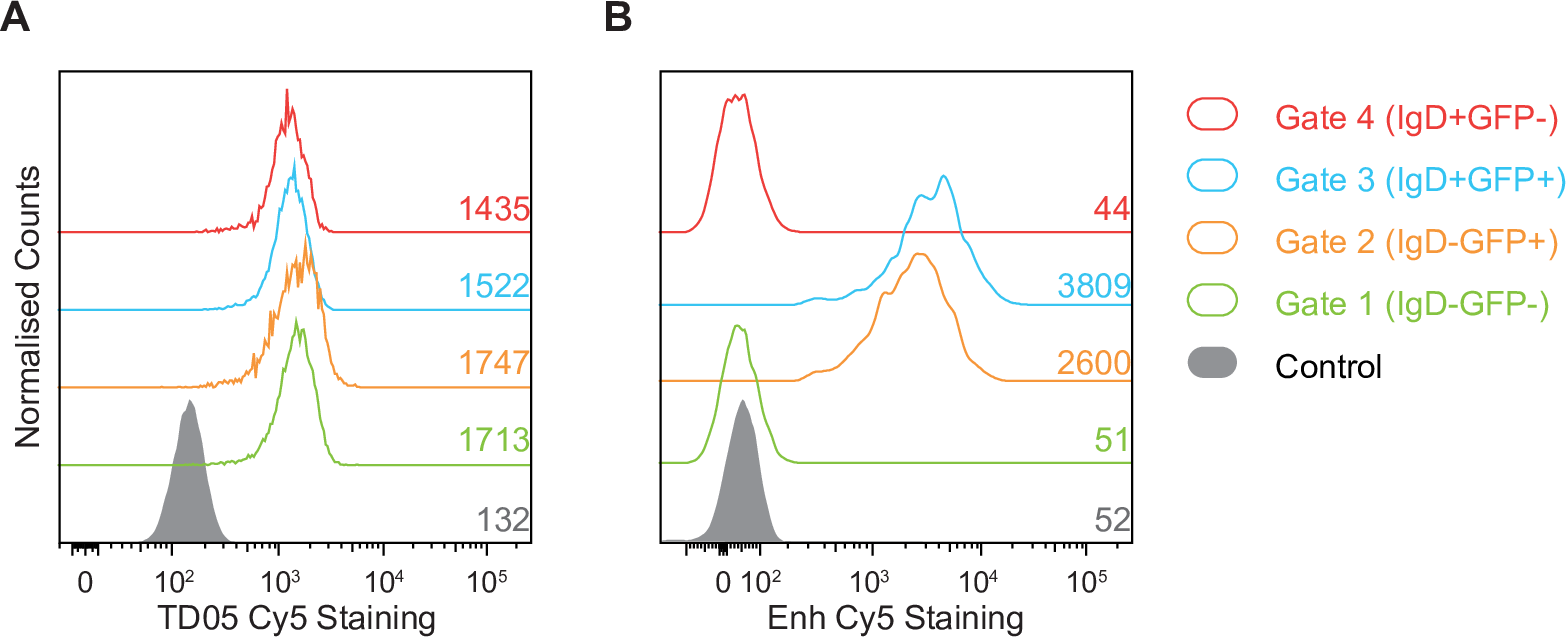

Supplement: S3 Fig — (A and B) Flow cytometry results showing the surface IgD- or IgM-BCR level evaluated by TD05 Cy5 staining (A) or GFP-μm level by Enh Cy5 staining (B) for the mixed Ramos cells following the gating strategy shown in Fig 3B. BCR, B cell antigen receptor; Cy5, cyanine 5; Enh, Enhancer; GFP, green fluorescent protein; IgD, immunoglobulin D; IgM, immunoglobulin M. (TIF) [file pbio.3000569.s003.tif]

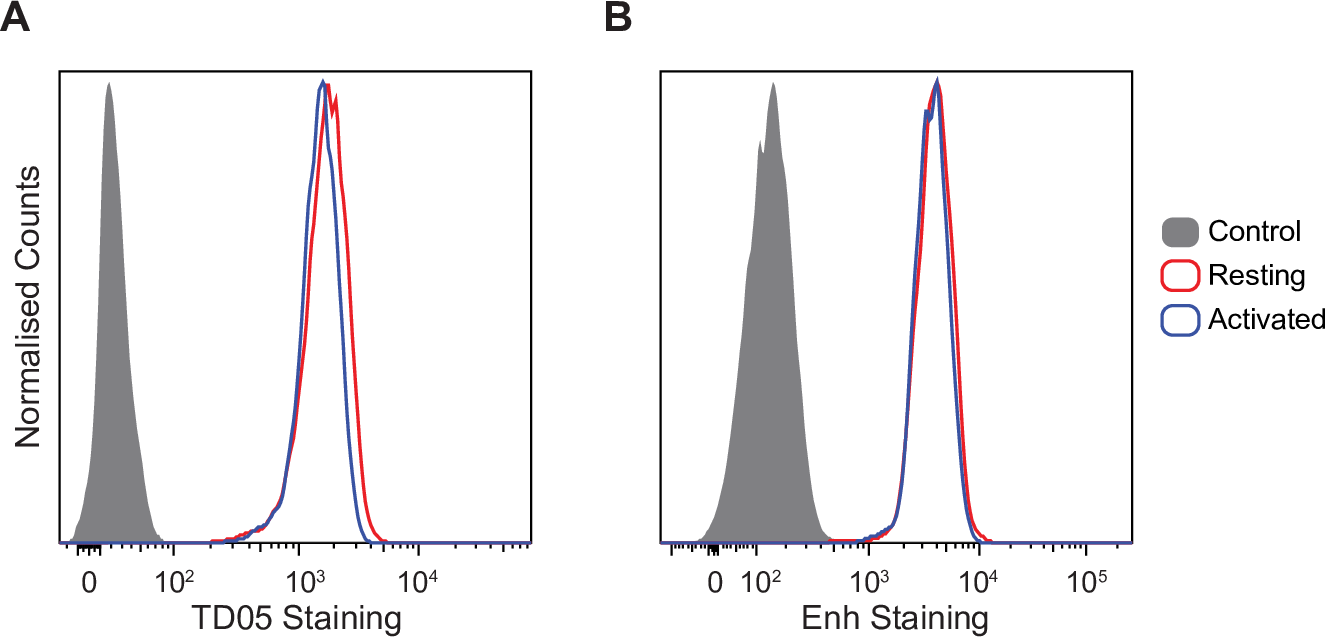

Supplement: S4 Fig — (A and B) Flow cytometry results showing the surface IgD-BCR level evaluated by TD05 Cy3 staining (A) or GFP-μm level by Enh Cy5 staining (B) for the resting and activated IgM-KO GFP-μm-expressing Ramos cells. BCR, B cell antigen receptor; Cy3, cyanine 3; Cy5, cyanine 5; Enh, Enhancer; GFP, green fluorescent protein; IgD, immunoglobulin D; IgM, immunoglobulin M; KO, knock-out. (TIF) [file pbio.3000569.s004.tif]

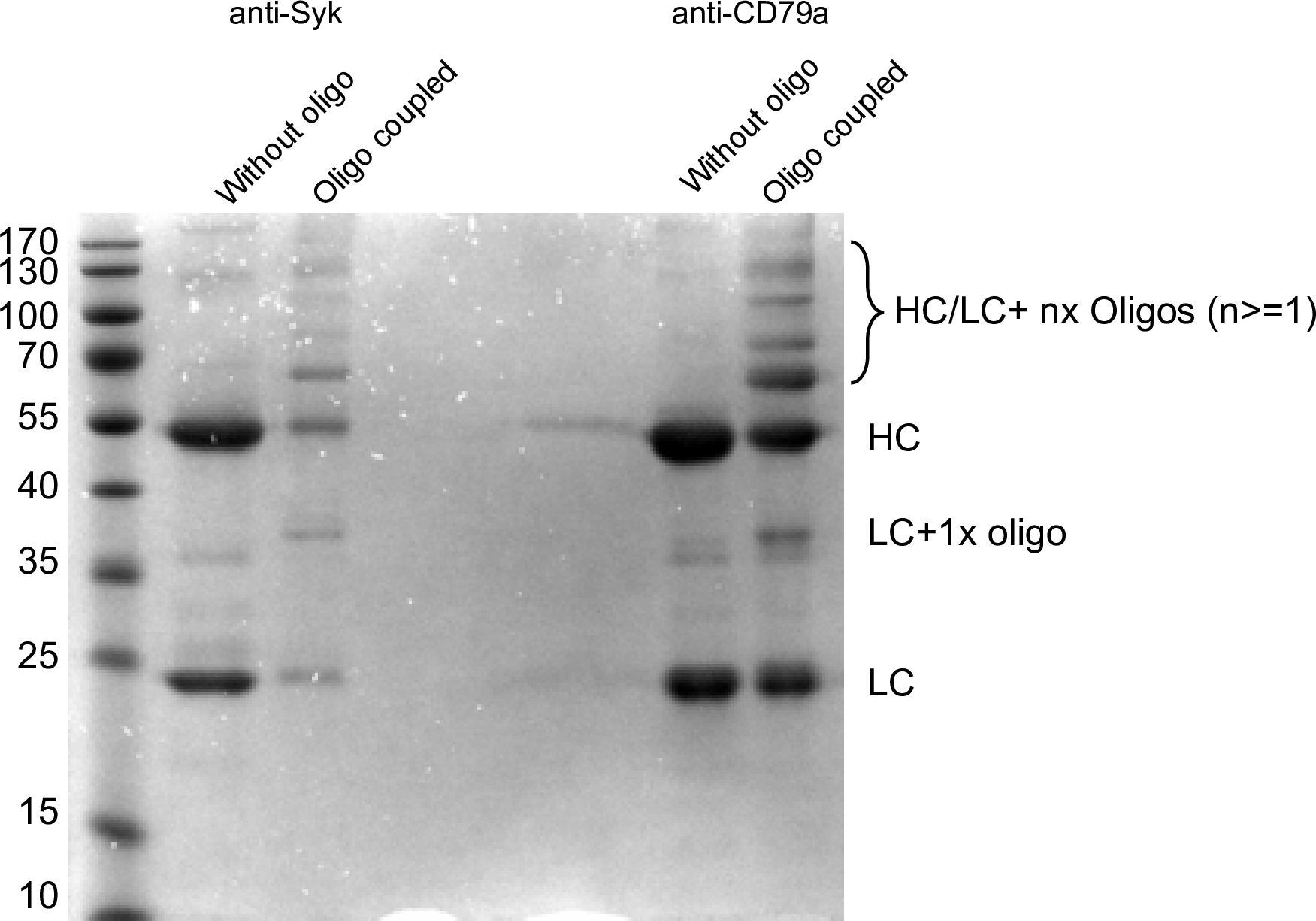

Supplement: S5 Fig — TGX; tris-glycine extended. (TIF) [file pbio.3000569.s005.tif]

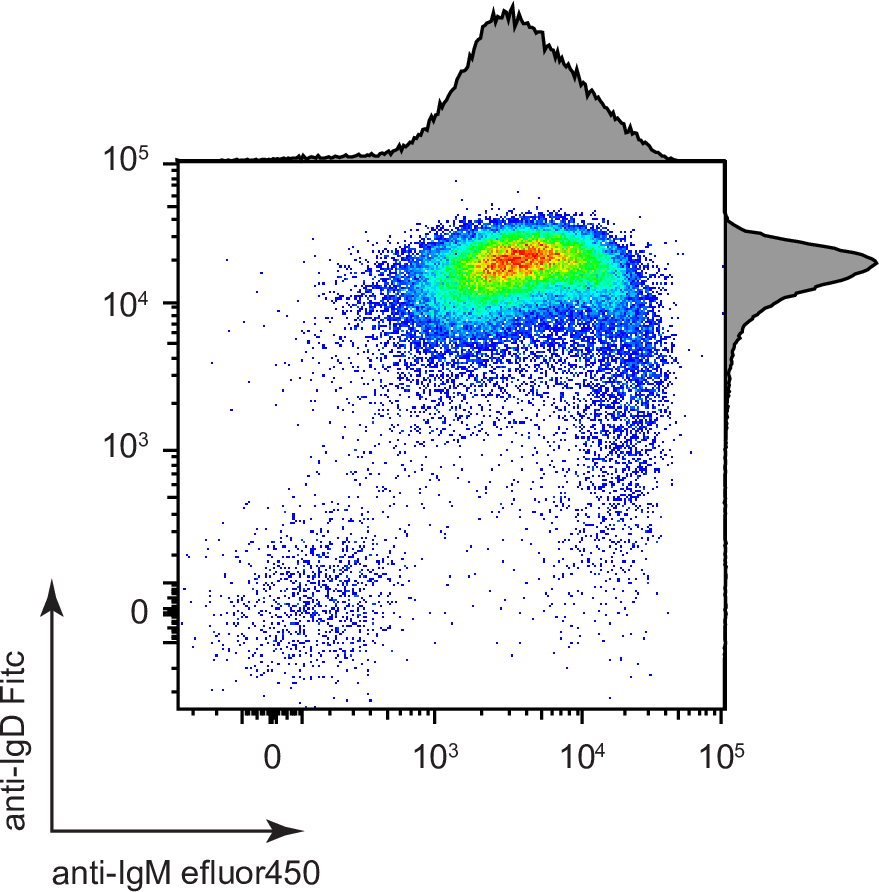

Supplement: S6 Fig — BCR, B cell antigen receptor; IgD, immunoglobulin D; IgM, immunoglobulin M. (TIF) [file pbio.3000569.s006.tif]
