# Supplementary material for: A new branched proximity hybridization assay for the quantification of nanoscale protein–protein proximity
Source: PLoS Biol. 2019 Dec 11;17(12):e3000569. doi: 10.1371/journal.pbio.3000569 (PMC6905527; doi:10.1371/journal.pbio.3000569)
Supplement: S1 Raw Images — (PDF) [file pbio.3000569.s009.pdf]

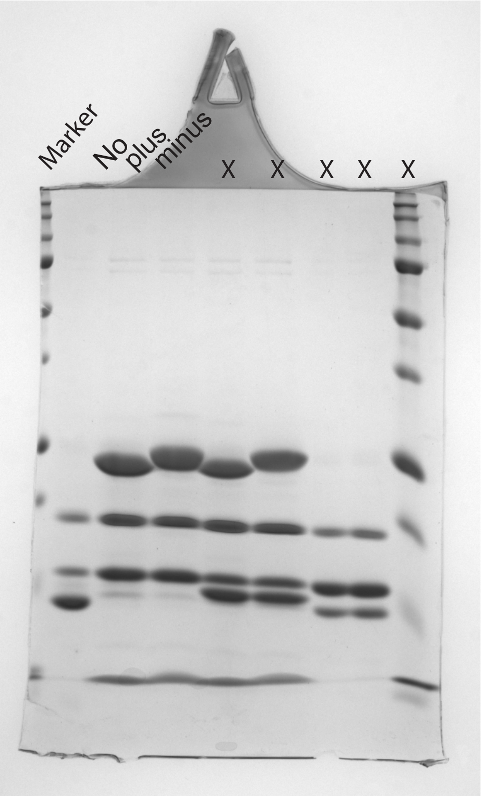

Raw image for S1B_Fig, Coomassie stained, image captured by Bio-Rad ChemiDoc Touch

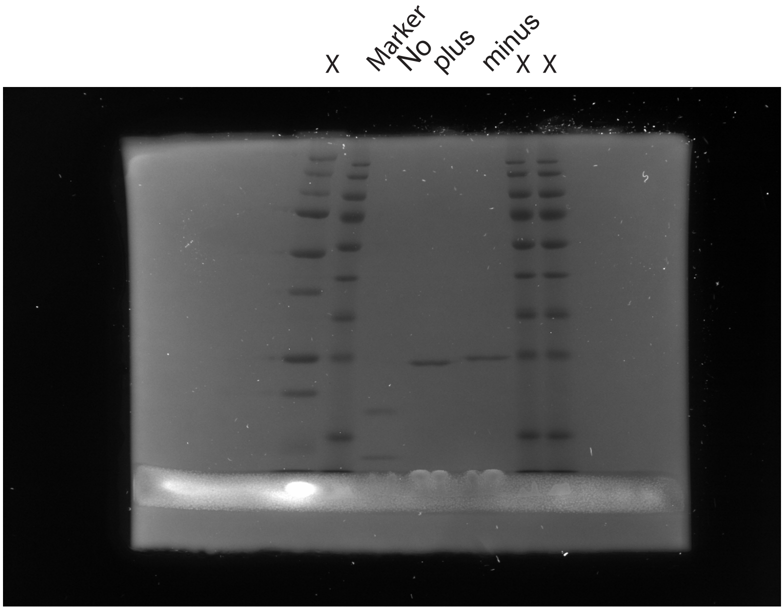

Raw image for S1C_Fig, Bia-Rad TGX Stain-Free gel, image captured by Bia-Rad Geldoc.

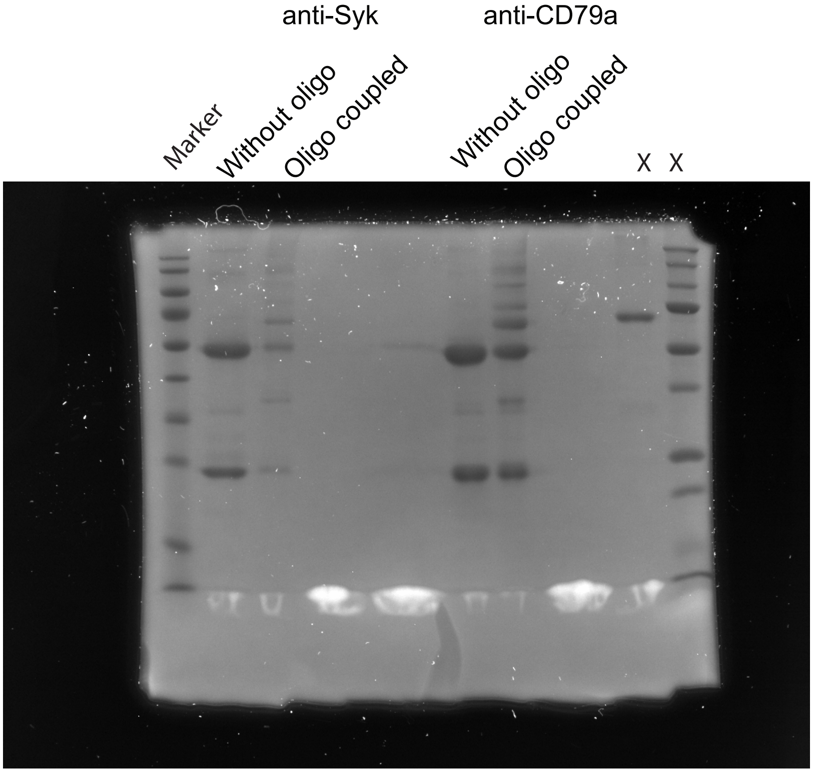

Raw image for S5_Fig, Bia-Rad TGX Stain-Free gel, image captured by Bia-Rad Geldoc.
