# Supplementary material for: A new branched proximity hybridization assay for the quantification of nanoscale protein–protein proximity
Source: PLoS Biol. 2019 Dec 11;17(12):e3000569. doi: 10.1371/journal.pbio.3000569 (PMC6905527; doi:10.1371/journal.pbio.3000569)
Supplement: Attachment — Submitted filename: Point to point answer.docx [file pbio.3000569.s010.docx]

Reviewer #1: The paper “A new branched proximity hybridization assay for the quantification of nanoscale protein-protein proximity” by Zheng et al. describes an approach to demonstrate colocation of proteins directly in biological samples. It is a matter of increasing importance to understand functional properties of cellular systems by observing how proteins may be interacting to exert their activities, and improved methods for their analysis can be of value.
The approach the authors describe is a variant of the widely used in situ PLA technique, commercially available from SigmaAldrich, and applicable both in situ and for flow cytometry as also shown by Zheng et al. using their bHPA technique. In both bHPA and in situ PLA samples are first treated with pairs of oligonucleotide-conjugated protein-binding reagents, such as antibodies. Zheng et al. demonstrate proximal binding by a hybridization-based local amplification technique called branched DNA, previously reported in reference 11 and here used via a kit, commercially available from ThermoFisher. By contrast, in the in situ PLA technique oligonucleotides on pairs of reagents brought in proximity serve as templates for ligation reactions that produce DNA circles. These DNA circles are then locally amplified via enzyme-catalyzed rolling circle amplification, resulting in prominent detection signals. 
Zhen et al. demonstrate the ability of their technique to reveal protein interactions in the context of signaling via the B cell antigen receptor on cell lines and mouse splenocytes, but the focus of the paper is on the method rather than the biology. I have some specific comments as follows:
1.	The branched DNA technique depends on that signals should only be detected when both members of the Z-DNA pairs have bound in proximity and can contribute to the strength of hybridization. In that regard it is of some concern that the bPHA signals illustrated by the light blue peak in Figure 1h are somewhat elevated over the background (shifted to the right), although still considerably lower than the red peak that illustrates the situation when all required reagents were present. This indicates that the “TD05-“ reagent alone can elicit some signal, something that could complicate analysis of protein interactions when target proteins are evaluated across a wide range of expression levels. The authors should comment on this, or preferably find conditions where this background is not observed.

*We thank the reviewer 1 for pointing this out. Indeed, we agree with reviewer 1 that the “TD05-“ reagent alone elicit some signal. We have modified the text for that. In addition, we have now performed similar controls with the Enh- reagent (see new Fig S2) and we could show that there is no such kind of signal. Together with the observation that TD05- only gives rather low signal comparing to the true bPHA signal, we would suggest that it is not a general problem for bPHA.*

2.	The reported 400-fold signal amplification (lines 16 and 68), should be supported either by referring to information about the kit or own measurements.

*The 400-fold signal amplification is based on the fact that each pre-amplifier contains 20 times the sequence for hybridizing with the amplifier and each amplifier contains 20 times the sequence for hybridizing with the fluorescent label probes. We now refer to the prime-flow manual for this information.*

3.	In the abstract the bHPA technique is said to offer better quantification of protein proximity (line 13), without mentioning what this is in comparison to. Elsewhere it is clear that the authors are comparing their approach to in situ PLA as is appropriate. Unfortunately, they do not provide any data from experimental side-by-side comparisons, however, making it difficult to evaluate to what extent their technique represents real progress. In situ PLA is said to result in a nonlinear amplification process (lines 42 and 194), but this claim is supported by reference 27, which represents a not fully relevant comparison between FRET and in situ PLA. On theoretical grounds it is not obvious why branched DNA amplification would yield a more linear result than rolling circle amplification, so such claims need to be supported by evidence from direct comparisons.

*We have now included in the manuscript a direct comparison between bPHA and PLA. (see new Fig.2)*

4.	The novel element of the paper is the use of branched DNA as an enzyme-free signal amplification method to reveal protein proximity via pairs of oligonucleotide-conjugated antibodies. It would therefore be relevant also to refer to and briefly discuss the paper “Proximity-dependent initiation of hybridization chain reaction” Bjorn Koos et al., 2015, DOI: 10.1038/ncomms8294. Like bHPA and in situ PLA this paper describes analysis of protein proximity via similar pairs of DNA-conjugated reagents but using another enzyme-independent local amplification technique namely a hybridization chain reaction.

*We thank the reviewer 1 for the information regarding the hybridization chain reaction-based proximity detecting method. We have now added a paragraph in the discussion part referring to this technique.*

5.	On lines 69 and 213 the claim is made that bHPA differs from in situ PLA in that in bHPA the oligonucleotides attached to the affinity reagents are not directly used for amplification, permitting multiplexing. This is a misunderstanding, however, as for in situ PLA secondarily added oligonucleotides are converted to a DNA circle used for amplification, and similar to bHPA this renders the assays suitable for multiplexing. (By contrast, in PLA used for protein detection in solution phase, the antibody-conjugated DNA strands are directly amplified via PCR)

*We have rephrased both parts to make it more clearer.*

6.	Since the purpose of the paper is to demonstrate the advantages of a local amplification method different from that used in the established in situ PLA technique for protein proximity analyses, it would be preferable if side-by-side experimental comparisons were presented to help the reader evaluate how the two procedures compare.  It would also be helpful to offer a theoretical comparison, perhaps in a table form, between bHPA, in situ PLA and the above mentioned proximity-dependent hybridization chain reaction. For example, the latter and bHPA differ from in situ PLA in that no enzyme is required for signal  amplification, potentially simplifying automation.

*We have now included in the manuscript a direct comparison between bPHA and PLA. (see new Fig.2). We also added a paragraph in the discussion part to compare the different methods.*

Reviewer #2: In the manuscript entitled ” A new branched proximity hybridization assay for the quantification of nanoscale protein-protein proximity” Zheng et al describes a method to probe for proximity, using branched hybridization to obtain a signal amplification. The authors show convincing data on the performance of the method. The advantage with the methods compared with methods such as PLA is that it does not require any enzymatic steps, hence should be more inexpensive. Novel methods for biology are needed and the development described herein will be of interest to the scientific community.

*We thank the reviewer 2 for this positive judgment.*

I only have a minor suggestions to the authors.

1.	The method section is rather short and it would be valuable if more details could be added, e.g. sequences of oligos used, buffer conditions for hybridization, where was sortase obtained? For a reader who want to replicate the experiments or use the method, it is important to provide all details regarding the method.

*We have now revised the materials and methods part by further providing the following information:*

*1) the sequences of the oligos used.*

*2) buffer conditions for hybridization*

*3) the origin of Sortase.*

*We hope that will allow readers to use bPHA for their application.*

2.	Why the intermediate step with hybridization of Z-DNA, can the pre-amplifier be hybridized directly to the minus and plus probe? That would remove one step of the assay.

*You are right, it Is not necessary to use the Z-DNA. Amplifying the proximity signal without Z-DNA indeed would remove one step of the assay. However, the use of Z-DNA makes thing easier under some situations. WE have now rephrased that part to make it clearer.*
