# Supplementary material for: A new branched proximity hybridization assay for the quantification of nanoscale protein–protein proximity
Source: PLoS Biol. 2019 Dec 11;17(12):e3000569. doi: 10.1371/journal.pbio.3000569 (PMC6905527; doi:10.1371/journal.pbio.3000569)
Supplement: Attachment — Submitted filename: Point to point answer.docx [file pbio.3000569.s011.docx]

Reviewer remarks:

Reviewer #1: Zheng et al. have now resubmitted their paper after addressing comments by both reviewers. 

As previously, the authors claim on lines 19 and 71 a 400-fold amplification by the bPHA technique, now supported by a reference to information from the manufacturer, but the number represents a theoretical maximal signal amplification, and since no evidence is given that this amplification is actually achieved it would be prudent to describe this as a theoretical limit. Comparing panels 2B and 2D the amplified bPHA signals, where detection depends on binding by pairs of affinity reagents, seems to yield around ten-fold higher fluorescence than that of a single, unamplified Enh-Cy5 probe. Admittedly, the dependence for detection on binding by pairs of probes might reduce efficiency compared to assays that depends on binding by single reagents so this may not be a fair comparison for estimating signal amplification.

*We were asked by the reviewer 1 in the first round of the revision to provide supports “either by referring to information about the kit or own measurements”. We came with the reference to information from the manufacturer. However, it seems that the reviewer 1 is not really satisfied with that. Since we agree with reviewer 1 that the 400 folds is only a theoretical maximal and we did not provide experimental proof for that, we have now modified our manuscript to make this clear.*

Throughout the manuscript the authors describe the RCA process as nonlinear and the bDNA techniques as linear, but it is unclear what they mean by this. Both RCA and bDNA would be expected to give rise to local amplification products in direct proportion to the proper starting molecules, in the case of RCA a DNA circle and for bPHA proximal target sequences for the two Z-DNA probes. In the new figure 2 GFP expression of the Ramos cells seems to vary over more than an order of magnitude in the bPHA experiment, but less than one order of magnitude for the PLA experiment, complicating estimation of correlation or linearity of responses.

*We agree with the reviewer 1 that “Both RCA and bDNA would be expected to give rise to local amplification products”. But we do not agree with reviewer 1 that the amplification is “in direct proportion to the proper starting molecules” for both of them. For RCA, the DNA circle is amplified by an enzymatic reaction and for bPHA it is amplified by the hybridization. This difference is clearly seen when Fig 2E and 2H is compared for their bPHA(or PLA)/GFP correlation. We admit that the recorded GFP signals were different for the bPHA and PLA experiments. It is because the cells were fixed following different protocols. We noticed a different increase of autofluorescence in several fluorescence channels including GFP for the two different fixing conditions. Thus, we have to use different settings to record the results of PLA and bPHA experiments even though the same batch of the cells were used and the two experiments were actually started at the same time. By this means, the results from these two experiments are not directly comparable. Luckily, we are not relying on the comparison of the measured fluorescence signals but rather how the bPHA or PLA signals are correlated with the GFP signals and our results clearly says that these two methods are not equal regarding the amplification of signal .*

In line 225 bPHA is said to correlate to GFP over three orders of magnitude differences of GFP expression, but this is not evident from figure 2 where in the bPHA experiment GFP varied over a little more than an order of magnitude. It would be prudent to point this out. 

*We thank the reviewer 1 for pointing out this problem. Indeed, in our data presented in Fig 2D and 2E, the GFP fluorescence values span from 424 to 8205, roughly 20 times. However, the bPHA values cover a range between 1889 and 91200, closing to three orders of magnitude. It seems that we mixed it up with the GFP. We have now corrected the corresponding part.*

This figure 2 is rendered a little confusing by the inclusion of zero values in the logarithmic axes of panels B, C, D, F and G. The zero values should be replaced by the proper numbers. This is also true for panels 1H and 3B,C and D, 4A and 5B, as well as for supplementary figures 2, 3, 4 and 6.

*It is true that a normal logarithmic axis shall not include 0 as well as minus values. Fluorescence intensity of cells measured by flow cytometer are normally distributed over wide dynamic range. Therefore, traditionally flow cytometer results are presented with logarithmic axis. However, flow cytometer measurements can also produce data with their values equal to or even lower than zero. It will be more appropriate to present these data with linear axis. To take the best of both the logarithmic and linear worlds, modern software such as FlowJo that we used for our data analysis, provide transforms like “Biexponential” to better present the data. As a result of that, the axis includes 0 and minus logarithmic scales together with positive logarithmic scales.*

As a final point, were the TD05+ and – probes really used at 1 mM concentration as stated in line 339? This is a surprisingly high concentration.

*We thank the reviewer 1 for pointing out this problem. It should be 1 micromolor. We originally used symbol fonts to make the micro symbol. However, it was changed to times new roman accidently and become milli. We have now corrected that.*

Reviewer #2: All points raised have been addressed.

*We thank the reviewer 2 for acknowledging our efforts in improving the manuscript.*
